# Restricting the distribution of visual attention reduces cybersickness

**DOI:** 10.1186/s41235-023-00466-1

**Published:** 2023-03-16

**Authors:** Sai Ho Yip, Jeffrey Allen Saunders

**Affiliations:** Department of Psychology, University of Hong Kong, Hong Kong, Hong Kong

**Keywords:** Cybersickness, Simulator sickness, Virtual reality, Visually induced motion sickness, Visual attention, Misery scale, SSQ, MSSQ

## Abstract

This study investigated whether increased attention to the central or peripheral visual field can reduce motion sickness in virtual reality (VR). A recent study found that increased attention to the periphery during vection was correlated with lower self-reported motion sickness susceptibility, which suggests that peripheral attention might be beneficial for avoiding cybersickness. We tested this experimentally by manipulating visual attention to central vs. peripheral fields during VR exposure. We also measured attention to the periphery during vection and motion sickness susceptibility to attempt to replicate the previous results. In Experiment 1, task-relevant cues to target locations were provided in the central or peripheral field during navigation in VR, and we found no differences in motion sickness. In Experiment 2, attention to the center or periphery was manipulated with a dot-probe task during passive VR exposure, and we found that motion sickness was greater in the condition that required attention to the periphery. In both experiments, there was no correlation between baseline attentional allocation and self-reported motion sickness susceptibility. Our results demonstrate that restricting attention to the central visual field can decrease cybersickness, which is consistent with previous findings that cybersickness is greater with large FOV.

## Significance statement

Some recent research suggests that allocating attention to the periphery may reduce motion sickness in virtual reality, but this has not been directly tested. We tested this experimentally by manipulating visual attention during virtual reality exposure and measuring motion sickness. Contrary to the hypothesized effect, we found that motion sickness was greater with peripheral attention. We also failed to replicate the previous correlational findings. Our findings potentially have both theoretical and practical implications.

## Introduction

Virtual reality (VR) exposure often results in visually induced motion sickness (VIMS) symptoms, such as oculomotor discomfort, eyestrain, and nausea (Golding, 1998; Weech et al., 2019). It has been estimated that around 61–80% of the population will experience mild to severe discomfort during VR interactions (Lawson, 2015). Motion sickness in VR has been termed “Cybersickness,” “Simulator Sickness,” or “Virtual Reality Sickness” (McCauley & Sharkey, 1992; Palmisano et al., 2017; Saredakis et al., 2020). For consistency purposes, we will adopt the term “Cybersickness” when referring to VIMS in VR.

According to the sensory conflict theory (Reason, 1978), cybersickness is the result of the sensory mismatch between the visual and non-visual systems, with the most prominent being the conflict between the visual and vestibular inputs. When users experience simulated movement in VR, they receive visual cues to self-motion without corresponding vestibular cues (Weech et al., 2019). It has been theorized that incongruent sensory cues can trigger a response to possible poisoning, leading to VIMS symptoms (Keshavarz & Hecht, 2012).

### Visual attention and cybersickness

Wei et al. (2018) proposed that reallocation of visual attention may help mitigate cybersickness and observed some supporting correlational evidence. They measured the degree of attention allocation to the central and peripheral visual fields during vection—a visually induced sense of self-motion that is commonly experienced during VR exposure (Weech et al., 2019)—and asked the subjects to report their motion sickness susceptibility using the Motion Sickness Susceptibility Questionnaire Short-form (MSSQ-Short). Wei et al. (2018) found that greater attention to the peripheral visual field (PVF) during vection was correlated with lower self-rated general susceptibility to motion sickness.

Wei et al. (2018) attributed their findings to the conflict-reducing mechanism proposed by Brandt et al., (1998, 2002). When experiencing the same level of sensory conflicts, different individuals may exhibit varying motion sickness severity based on their ability to regulate visual and vestibular inputs. Those who exhibit fewer VIMS symptoms when experiencing vection possess the ability to downregulate vestibular inputs, while simultaneously strengthening the visual system signals.

It has been hypothesized that people tend to rely on their visual periphery more than the central visual field (CVF) inputs to determine self-motion (Dichgans & Brandt, 1978; Ungerleider, 1994). Wei et al. (2018) argue that increased attention to the PVF will make visual simulations of self-motion more convincing and strengthen the perception of self-motion. Increased attention to visual information about self-motion could also reduce attention to vestibular inputs. The combination of strengthening the reliability of visual motion information and downplaying vestibular signals might help people to minimize the visual–vestibular conflict experienced during vection.

### Field of view and cybersickness

The idea that more attention to the visual periphery reduces cybersickness appears to conflict with the results of other studies that have found that a large field of view (FOV) increases cybersickness (Lin et al., 2002; Rebenitsch & Owen, 2016; Saredakis et al., 2020; Seay et al., 2002; Weech et al., 2019). Restricting the FOV removes peripheral motion information and likely results in a narrower range of attention. If attention to the peripheral motion helps to reduce cybersickness, a small FOV would be expected to produce more cybersickness, which is contrary to the empirical findings.

A possible explanation for the effect of FOV on cybersickness is that peripheral motion information increases the visual–vestibular conflict that arises when a stationary observer is presented with simulated self-motion (Weech et al., 2019). With large FOV, the visual cues to self-motion would be stronger due to the peripheral input, so there would be more conflict with non-visual cues that specify that the observer is stationary.

This explanation for FOV effects suggests that increased attention to the CVF, rather than the PVF, would reduce cybersickness. Forcing VR users to restrict their attention to their CVF might decrease the contribution of peripheral motion information, similar to restricting FOV, and thereby reduce cybersickness.

However, restricting attention to the CVF may not be equivalent to removing peripheral information entirely, and attention allocation might affect cybersickness in other ways. It remains possible that removing peripheral motion cues can reduce motion sickness in VR, but increased attention to the visual periphery can also mitigate cybersickness.

### Current study: manipulating visual attention during a VR task

Although the results of Wei et al. (2018) suggest that visual attention to the PVF can reduce cybersickness, there are some limitations to their study that make it difficult to draw a strong conclusion. First, Wei et al. (2018) only measured self-reported susceptibility to motion sickness and did not measure actual motion sickness experienced during vection. Second, Wei et al. (2018) did not manipulate their subjects’ visual attention, so the relationship between visual attention and VIMS susceptibility was merely correlational. To demonstrate a causal relationship between visual attention and cybersickness, it would be necessary to manipulate subjects’ visual attention allocation when performing a VR task.

The present study tested the relationship between attention allocation and cybersickness by manipulating visual attention during VR exposure and directly measuring experienced cybersickness. Subjects performed a navigation task (Experiment 1) or passively viewed simulated motion (Experiment 2) in conditions that were expected to produce some motion sickness symptoms. Separate conditions were designed to encourage attention to either central or peripheral vision, and subjects rated their motion sickness using standard subjective measures.

In Experiment 1, we manipulated visual attention by presenting task-relevant visual cues in either the peripheral or central regions of the displays. In the peripheral-cued condition, targets were highlighted when they were in the outer periphery of the visual displays, which is expected to increase attention to the PVF. In the central-cued condition, targets were highlighted only when in the center of the visual displays, which would tend to increase attention to the CVF. The visual information about self-motion would not be affected by the highlighting of targets, so any effects on cybersickness could be attributed to differences in attentional allocation. A drawback of this approach is that it is hard to control fixation. In Experiment 2, we manipulated attention by superimposing a dot-probe task during exposure to provide a stronger manipulation.

We also attempted to replicate the finding by Wei et al. (2018) that attentional allocation during vection is correlated with general motion sickness susceptibility. In addition to the VR exposure task, subjects completed the Motion Sickness Susceptibility Questionnaire Short-form (MSSQ-Short) to measure their VIMS susceptibility and performed the Sustained Attention to Response Task (SART) based on Wei et al. (2018). SART is a reaction task that can measure the relative amount of attention allocated to CVF and PVF during vection.

If heightened attention to the periphery during simulated motion decreases cybersickness, as proposed by Wei et al. (2018), this would predict:

- H1: For the virtual reality exposure conditions, subjects in the peripheral-cued condition would experience less cybersickness.
- H2: Individual differences in performance for PVF vs CVF stimuli in the SART task would be negatively correlated with self-reported motion sickness susceptibility, with increased attention to PVF associated with a decreased tendency toward motion sickness.
- H3: Individual differences in SART performance would also be negatively correlated with the overall cybersickness experienced in the VR exposure conditions.

Alternatively, attending to the periphery during simulated motion might produce more cybersickness than attending to the central visual field. This would be consistent with findings that cybersickness increases with larger FOVs (Lin et al., 2002; Rebenitsch & Owen, 2016; Saredakis et al., 2020; Weech et al., 2019). If so, this would predict:

- H1a: Subjects in the peripheral-cued exposure condition would experience more cybersickness.
- H2a: Individual differences in performance for PVF vs CVF stimuli would be positively correlated with motion sickness susceptibility, with increased attention to PVF associated with a greater tendency toward motion sickness.
- H3a: Individual differences in SART performance would also be positively correlated with the overall cybersickness experienced in the VR exposure conditions.

## Experiment 1

In Experiment 1, we tested whether allocating attention to the peripheral visual field during virtual navigation reduces the severity of cybersickness. Attention was manipulated by presenting task-relevant visual cues in either the peripheral or central regions of the display, and motion sickness in peripheral and central conditions was compared. In addition to the virtual navigation task, subjects performed the SART task and reported their motion sickness susceptibility to test whether we could replicate the correlation observed by Wei et al. (2018).

## Methods

### Preregistration

The methods and analysis plan were preregistered on the Open Science Framework (OSF) before data collection: https://osf.io/rfzwt. One deviation from the preregistration is that we used the median response times, estimated by the Hodges–Lehmann estimator, rather than mean response times for our analysis of the SART results. We used the robust measure to reduce the influence of outliers in response times. This choice did not change any qualitative results. In the preregistration, we stated that the SSQ scores would be transformed by log_10_(SSQ + 10). We instead used an equivalent transform log_10_(SSQ/10 + 1). The results are identical except for a constant shift, which has no effect on statistical test results. We also performed some exploratory analyses in Experiment 1 that were not preregistered. There were no other deviations from the preregistered plan.

### Participants

Sixteen people participated in this experiment. The sample consisted of 7 males and 9 females, with ages between 21 and 24 years old (*M* = 22.4, *SD* = 1.09). Subjects were recruited primarily through personal connections and were paid $200 HKD as compensation. All subjects reported that they had normal or corrected-to-normal vision, and no history of impairment in vestibular or neurological functioning.

The sample size was determined by a sequential procedure, shown in Table 1. The interim test used to determine stopping was a t test comparing the MISC ratings in the peripheral-cued and central-cued conditions. The stopping criteria were designed to keep the overall Type I error rate less than 5% with no adjustment of the test alpha (i.e., the standard alpha of .05 is used to determine statistical significance). Allowing the procedure to stop early when an interim test finds a significant difference would increase the overall Type I rate, but allowing the procedure to stop early when an interim test finds no difference would decrease the overall Type I rate. The stopping criteria were chosen to balance these two effects. The procedure is similar to some previously proposed methods that use *p* values from interim tests to determine stopping: the composite-limited-adaptive-sequential test procedure (Botella et al., 2006), and the variable-criteria sequential stopping rule procedure (Fitts, 2010).

**Table 1:** The sequential procedure used to determine sample size

Interim *N*	*p* value	Action and interpretation
*n* = 16	*p* < .01	Stop, significant difference
	*p* > .30	Stop, no significant difference
	.01 ≤ *p* ≤ .30	Continue until *n* = 24
*n* = 24	*p* < .02	Stop, significant difference
	*p* > .20	Stop, no significant difference
	.02 ≤ *p* ≤ .20	Continue until *n* = 32
*n* = 32	*p* < .05	Stop, significant difference
	*p* ≥ .05	Stop, no significant difference

The procedure was designed to detect a medium-to-large effect size with high efficiency. We had no prior basis for estimating the effect size for the experimental manipulation, and we planned to do a follow-up experiment to investigate any effects or trends that were observed in Experiment 1. The power and false-positive rate of the procedure were estimated by performing simulations, with 10^8^ simulations per case. For the null case, simulation results confirmed that the overall Type I rate was less than 5%, with an average *n* = 19.3. Simulations of nonzero effects found that the procedure has 94% power to detect an effect with *d*_*z*_ = 0.7, with an average *n* = 20.9, and 72% power to detect an effect with *d*_*z*_ = 0.5, with an average *n* = 23.1. The power as a function of effect size is approximately the same as using a fixed sample size of *n* = 28. In Experiment 1, the procedure stopped at the first interim stage (*p* = .608) with a sample size of *n* = 16.

### Apparatus and stimuli

Experiment 1 consisted of two tasks, the virtual navigation task and the Sustained Attention to Response Task (SART) based on Wei et al. (2018).

#### Virtual navigation task

For the virtual navigation task, subjects “moved” through virtual environments with scattered treasure chests. They were instructed to collect as many treasure chests as possible within a set amount of time (8 min). The chests were partially hidden inside bushes but were highlighted in red whenever they are in either the center or periphery of the display, depending on the experimental condition.

The virtual environments used in this task were coded with Unity version 2018.3.14f1 and presented using an HTC Vive head-mounted display (HMD). The scenes were rendered at a refresh rate of 90 Hz, with a resolution of 1080 × 1200 per eye (2160 × 1200 combined). The software field of view (FOV) was set to match the field of view of the displays. Heading movement was tracked at 90 Hz and used to continuously update the simulated views. Simulated movement through the environment was controlled using the HTC Vive controller touchpad. The upper and lower parts of the trackpad were used to control forward and backward simulated movement, and the left and right parts of the trackpad were used to control simulated rotation. The forward/backward speed was 6 m/s, and the rotation rate was 40°/s.

**Fig. 1:**
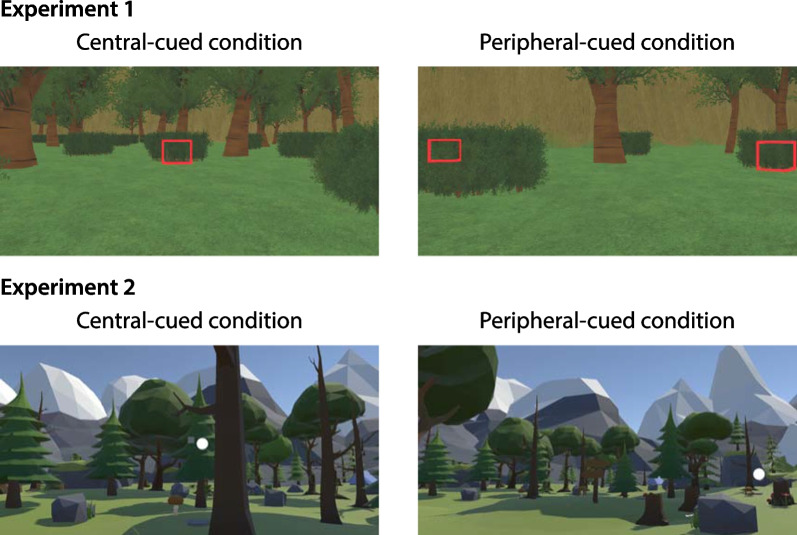
Screenshots from the central-cued and peripheral-cued conditions of Experiment 1 (top) and Experiment 2 (bottom). In Experiment 1, subjects navigated in a virtual environment to find targets chests that were obscured by bushes. The chests were sometimes highlighted in red depending on their position and the attentional cueing condition. In the central-cued condition (top left), targets were highlighted when their direction was within 10° of the central direction and less than 50 m away. In the peripheral-cued condition (top right), targets were highlighted when they were at least 40° to the left or right of the center and less than 50 m away. In Experiment 2, subjects passively viewed simulated movement through an environment while performing dot-probe task. In the central-cued condition (bottom left), the superimposed dots appeared within 5° of the center of the display. In the peripheral-cued condition (bottom right), the dots appeared in an annulus between 20 and 30° from the center

Two attention allocation conditions were tested in a within-subject design (Fig. 1–upper row). In the central-cued condition, the chests were highlighted when they are located inside a 10° diameter cone around the forward direction of the head. In addition, subjects were verbally encouraged to pay more attention to the CVF. A white transparent reference point was provided, which allowed the subjects to know where the center of the display was. For the peripheral-cued condition, the chests were only highlighted when they are located more than 40° away from the center measured horizontally, and subjects were told to attend more to their PVF. For both attention allocation conditions, chests were only highlighted when they were within 50 m of the player.

To prevent subjects from remembering the chest locations across conditions, two similar virtual forests were constructed. The only difference between the forests was the specific locations of the trees, bushes, and chests. Each virtual forest consisted of 150 empty bushes, 39 bushes with a chest, and 185 trees. The order of the two attention allocation conditions and pairing with the two virtual forests were fully counterbalanced.

#### Sustained Attention to Response Task (SART)

**Fig. 2:**
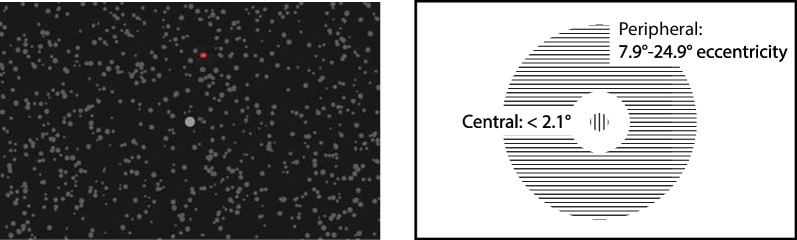
The Sustained Attention to Response Task (SART). Subjects fixated at a center point and a field of random dots rotated around the center. At random intervals, a red/green dot would appear either in the central region or the peripheral region

For the Sustained Attention to Response Task (SART), subjects fixated on a central point and responded whenever a dot of a certain color appeared. During the SART task, subjects were presented with a rotating dot pattern to invoke vection (Fig. 2). The pattern consisted of 600–650 gray dots on a black background that rotated anticlockwise at 32° per second. An additional gray dot located at the center of the screen served as the fixation marker, occupying 1.2° of the subjects’ FOV. The displays were presented with a BenQ HT4050 DLP Projector and a back-projection screen. The viewing distance was set to 72 cm, and the FOV of the projection area was 93.5° × 61.8°. The refresh rate was 60 Hz.

Static green/red dots either appeared inside the CVF region (FOV: 0°–2.1°) or the PVF region (FOV: 7.9°–24.9°) of the projection. Each colored dot was one SART trial. Subjects were required to respond to the red dots and ignore the green dots. The response time to each red dot was recorded and used for data analysis. The colored dots were presented for a duration of 500 ms, and the inter-trial interval varied between 1000 and 1500 ms. There were 200 SART trials in total, split between the two sessions. In each session, the SART trials were divided into four different types (10 × Green-CVF, 10 × Green-PVF, 40 × Red-CVF, 40 × Red-PVF) and presented in a randomized order.

### Measurements

#### Misery scale (MISC)

The Misery Scale (Bos et al., 2005) served as the primary indicator of cybersickness experienced during virtual reality exposure. It is a fast single-item instrument that monitors the course of cybersickness, without distracting the subjects from the virtual experience (Chang et al., 2020). The experimenter prompted the subjects to orally report a MISC score every 2 min of VR exposure. A MISC score can range from 0 to 10, with 0 indicating no discomfort, 6 suggesting a slight sense of nausea, and 10 representing vomiting.

The MISC scores used for the main analyses were from one of the four exposure blocks, selected based on completion rate. We expected that some subjects would not be able to complete all four exposure blocks. To ensure that the subjects only experienced mild cybersickness, the virtual navigation task was immediately stopped whenever symptoms were rated as 6 or more on the MISC scale. If only a small number of participants were unable to complete all four exposure blocks (< 25% of total subjects), we planned to use the MISC scores from the last completed blocks for analysis. However, if a larger number of subjects were unable to reach the final block (25% or more of total subjects), MISC scores from an earlier block would be used instead. We planned to select the last block that at least 75% of subjects were able to complete and use the MISC scores from that block for analysis, or the MISC scores from the final completed block for subjects who stopped earlier than the selected block.

#### Simulator Sickness Questionnaire (SSQ)

The Simulator Sickness Questionnaire (Kennedy et al., 1993) was our secondary measurement of experienced cybersickness. This self-report questionnaire is widely used as a subjective indicator of cybersickness (Chang et al., 2020; Weech et al., 2019). We administered the SSQ to subjects immediately before and after VR exposure. The SSQ consists of 16 items, with each question capturing the severity of a particular motion sickness symptom (e.g., headache, nausea) on a scale of 0–3. Responses were summed to obtain an SSQ total score, and scores for the nausea, oculomotor, and disorientation subscales.

The SSQ scores were transformed and normalized before performing statistical analysis. First, a log transform was applied to the raw SSQ scores, log_10_(SSQ/10 + 1), to reduce the skew of distributions. We further normalized the SSQ scores to compensate for any pre-exposure symptoms by subtracting the pre-exposure log-transformed SSQ scores from the post-exposure log-transformed SSQ scores.

#### Motion sickness susceptibility questionnaire short-form (MSSQ-short)

As in Wei et al. (2018), we used the Motion Sickness Susceptibility Questionnaire Short-form (Golding, 1998) to measure subjects’ general susceptibility to motion sickness. This self-report questionnaire consisted of 18 items. Each item measured the frequency of motion sickness (i.e., sickness and nausea) when exposed to a particular type of transportation/entertainment (e.g., small boat, roundabout) on a scale of 0–3. The MSSQ can generate a total score, child and adult subscale scores. The child subscore indicates the motion sickness susceptibility level before the age of 12, while the adult subscore represents the susceptibility over the last 10 years.

#### Attention allocation tendency

The attention allocation tendency of individuals during the SART task was quantified using the reaction times to CVF and PVF stimuli. We first computed the median response times to CVF and PVF stimuli using the Hodges–Lehmann estimator, which is a robust estimator of central tendency that is more efficient than the sample median (Hodges & Lehmann, 1963). We then computed the difference between response times to CVF and PVF stimuli, ΔRT = RT_CVF_ – RT_PVF_. Higher values of ΔRT indicate relatively more attention to the PVF during vection.

### Procedure

This study was a two-day experiment. At the beginning of the first session, we administered the Motion Sickness Susceptibility Questionnaire Short-form (MSSQ-Short). Subjects then performed a block of SART trials. After finishing the SART task, subjects were given a break to recover from any motion sickness symptoms. They then completed the pre-test Simulator Sickness Questionnaire (SSQ) and Misery Scale (MISC), followed by the virtual reality navigation task. The experimenter helped the subject put on the HMD and ensured that it was securely attached and centered. After they completed the virtual navigation, subjects completed the post-test SSQ. The second experimental session was the same as the first session except that the MSSQ-Short was not administered.

For the SART task, subjects were instructed to keep their eyes on the fixation marker and press the “Shift” key whenever a red dot appeared on the screen. A SART block included 100 trials presented over a period of about 4 min. We limited the duration of the SART task to avoid causing motion sickness symptoms.

For the navigation task, subjects were given 8 min to “collect" treasure chests scattered in the forest. Treasure chests were collected by moving close to the chests, which triggered an animation of the chest opening. Subjects remained seated when performing the virtual navigation. To manipulate visual attention, central or peripheral cues (i.e., chests being highlighted with a red outline) were provided depending on the experimental condition. Every two minutes during the exposure, subjects were asked to verbally report their MISC rating of cybersickness symptoms. The virtual task was terminated immediately whenever the subjects reported a MISC score ≥ 6, or by their request due to significant discomfort.

Before performing the virtual navigation, subjects performed a short practice block to familiarize themselves with the navigation and task. The virtual environment for practice was a single room with four treasure chests. Subjects moved to collect the four treasure chests before proceeding to the main navigation task.

## Results

### Cybersickness during VR Exposure

#### Virtual navigation completion rate

**Fig. 3:**
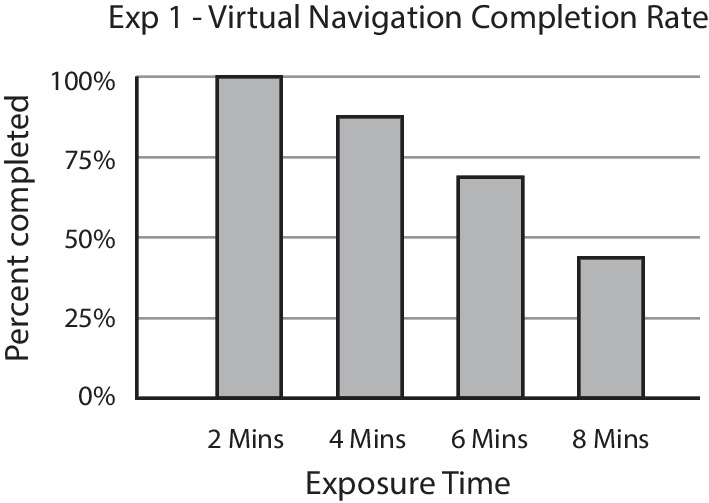
Percent of subjects who completed each of the virtual navigation blocks in Experiment 1. The procedure was stopped if subjects reported more than mild motion sickness symptoms (>6 on MISC scale). Less than half of the subjects were able to complete the full 8 min of exposure

Before comparing the cybersickness in the two conditions, we determined the exposure block to be used for the analysis of MISC scores, following our preregistered plan. Figure 3 shows the percentage of subjects that were able to complete each of the four blocks. Only 43.75% of the participants (*n* = 7) were able to complete all four exposure blocks. The last block that was completed by at least 75% of subjects was the 4-min block (87.5%, *n* = 14). Therefore, we used the MISC scores from the 4-min block for all subjects that completed the second block, and the MISC scores from the 2-min block for those who stopped earlier.

#### Central-cued vs. peripheral-cued conditions (H1 and H1a)

To test the main hypothesis of this study (H1 and H1a), we conducted paired-sample t tests to compare the mean cybersickness scores (MISC and SSQ) in the two attention allocation conditions. Statistical tests were conducted on the raw MISC scores and the normalized and transformed SSQ scores.

**Fig. 4:**
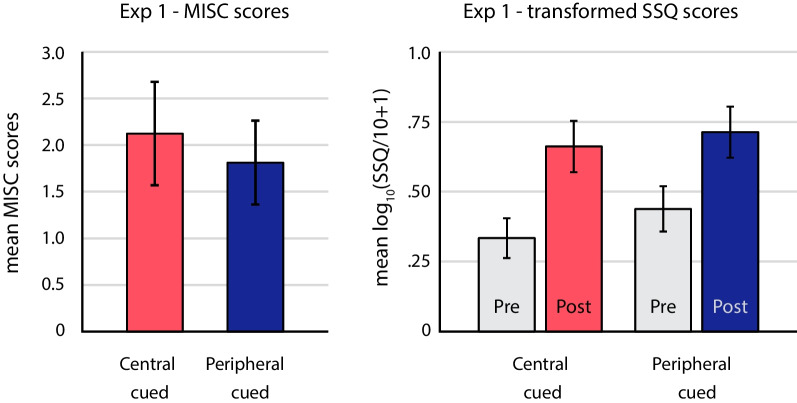
Motion sickness in the central-cued (red) vs peripheral-cued (blue) conditions of Experiment 1. The left graph plots mean MISC scores. The right graph plots mean SSQ scores after transforming by log_10_(SSQ/10 + 1). Pre-exposure SSQ scores are shown in gray. Error bars depict standard errors of the mean

The results did not reveal any reliable difference between the cybersickness induced in the central-cued and peripheral-cued conditions. Figure 4 plots the mean MISC and SSQ scores for the two attention allocation conditions. There was a trend toward less motion sickness in the peripheral-cued condition, but this trend was not statistically significant for either motion sickness measure. There was no significant difference between the mean MISC scores in the central-cued condition (*M* = 2.125, *SD* = 2.217) and the peripheral-cued condition (*M* = 1.813, *SD* = 1.797), *t*(15) = 0.524, *p* = .608, *d*_*z*_ = 0.131. Similarly, for the SSQ scores, there was no significant difference between the change in log_10_(SSQ-T/10 + 1) in the central-cued condition (*M* = 0.328, *SD* = 0.196) and in peripheral-cued condition (*M* = 0.276, *SD* = 0.283), *t*(15) = 0.635, *p* = .535, *d*_*z*_ = 0.159. We also compared results from the SSQ subscales and found no differences between the attentional allocation conditions (nausea: *t*(15) = 0.544, *p* = .595, *d*_*z*_ = 0.136; oculomotor disturbances: *t*(15) = 0.137, *p* = .893, *d*_*z*_ = 0.034; disorientation: *t*(15) = 1.249, *p* = .231, *d*_*z*_ = 0.312). Overall, our results provide no evidence that the attention manipulation affected cybersickness.

### Attention allocation tendency during vection

#### Attention allocation tendency and MSSQ-short (H2 and H2a)

**Fig. 5:**
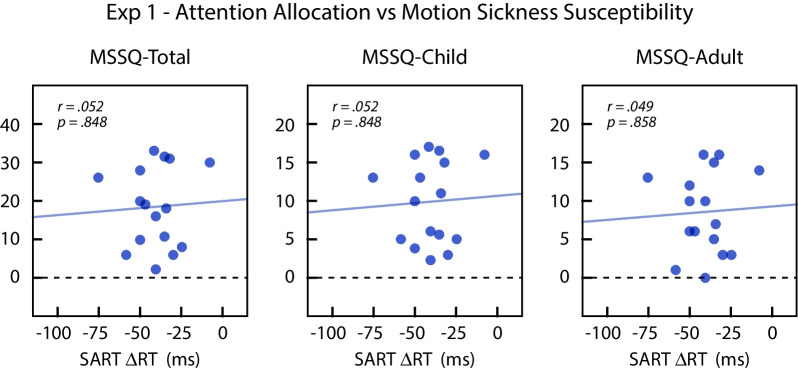
Self-reported motion sickness susceptibility (MSSQ) of individual subjects in Experiment 1 plotted as a function of their attentional allocation scores derived from SART. The attentional allocation score is the mean difference in reaction times for peripheral and central targets: ΔRT = RTCVF − RTPVF. Positive values of ΔRT correspond to more attention to the periphery. Lines show the regression fits. The left graph plots the total MSSQ scores, and the middle and right graphs plot the MSSQ child and MSSQ adult subscores

We performed correlation analyses to test the relationship between attention allocation tendency during the SART task and self-reported motion sickness susceptibility (H2 and H2a), as measured by the MSSQ-Short total scores and the two MSSQ-Short subscale scores (child and adult). Based on the findings of Wei et al. (2018), increased attention to the PVF was expected to be associated with lower self-reported motion sickness susceptibility. This was not observed. Figure 5 plots the score from the three MSSQ-Short measures as a function of the attentional allocation score (ΔRT). We did not find a significant correlation between attention allocation and the MSSQ-Short total scores (*r*(14) = .052, *p* = .848), MSSQ-Short child subscores (*r*(14) = .052, *p* = .848), or MSSQ-Short adult subscores (*r*(14) = .049, *p* = .858). The results showed no evidence for a linear relationship between attention allocation tendency during vection and self-reported motion sickness susceptibility scores.

#### Attention allocation tendency and cybersickness (H3 and H3a)

We performed exploratory analyses to examine the relationship between attention allocation tendency during vection and the overall cybersickness experienced in the virtual navigation task (H3 and H3a). Measures of the overall cybersickness level for each subject were computed by averaging the post-exposure MISC or SSQ scores from the central-cued and peripheral-cued conditions.

**Fig. 6:**
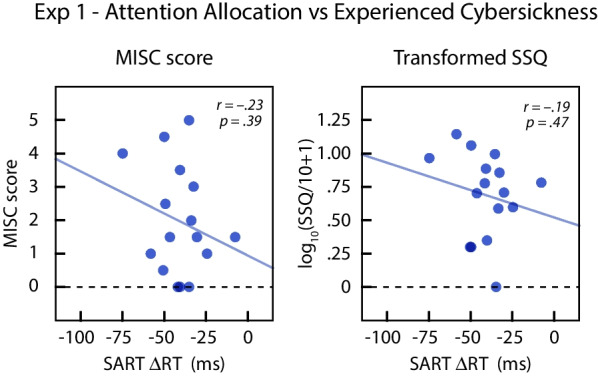
Cybersickness experienced by individual subjects during virtual navigation in Experiment 1 plotted as a function of their attentional allocation scores derived from SART (ΔRT = RT_CVF_ − RT_PVF_). Positive values of ΔRT correspond to more attention to the periphery. Lines show the regression fits. The left graph plots the MISC scores and the right graph plots the SSQ scores after transforming by log_10_(SSQ/10 + 1)

We did not detect a correlation between attention allocation scores (ΔRT) and the MISC scores (*r*(14) = − .233, *p* = .385) or the SSQ scores (*r*(14) = − .194, *p* = .470). Figure 6 plots the two cybersickness measures as a function of the attention allocation scores. Although there was a negative trend for both MISC and SSQ scores, neither showed a statistically significant relation to baseline attention allocation tendency.

### MSSQ-short and cybersickness

**Fig. 7:**
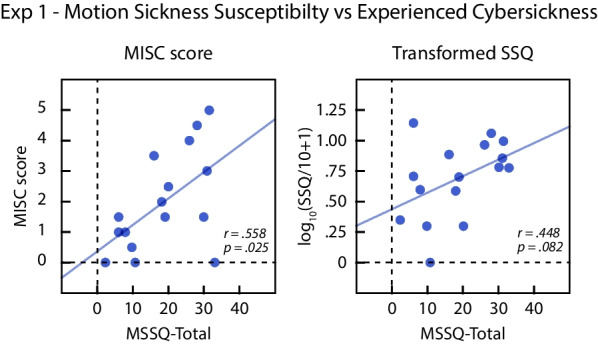
Cybersickness experienced by individual subjects in Experiment 1 plotted as a function of self-reported motion sickness susceptibility (MSSQ total). Lines show the regression fits. The left graph plots the MISC scores and right graph plots the SSQ after transforming by log_10_(SSQ/10 + 1)

We performed additional exploratory analyses to test whether MSSQ scores are predictive of experienced cybersickness. Figure 7 plots the two measures of overall cybersickness as a function of MSSQ-Short total scores. There was a significant positive correlation between MSSQ-Short total scores and overall MISC scores, *r*(14) = .558, *p* = .025, and a non-significant trend in the same direction for the SSQ scores, *r*(14) = .448, *p* = .082. Overall, our results suggest that MSSQ-Short scores are moderately predictive of cybersickness in our virtual navigation task.

## Discussion

### Manipulation of visual attention and cybersickness

We found no difference between motion sickness ratings in the two attention allocation conditions, using either the MISC or SSQ scores. There was a small trend toward lower cybersickness in the peripheral-cued condition, but it was not statistically significant. Our findings do not provide support for the hypothesis that more attention to the PVF will alleviate cybersickness level.

However, our conclusions are limited by the fact that the peripheral-cueing effect may have been temporary. For the peripheral-cued condition, it is possible that our subjects quickly shifted their fixation to the cued location whenever a chest was highlighted. If so, attention may have only been briefly reallocated to the periphery in this condition. In Experiment 2, we addressed this issue by implementing a stronger manipulation of visual attention allocation during virtual reality exposure.

### Visual attention allocation and motion sickness susceptibility

We were not able to replicate the results reported by Wei et al. (2018). We did not observe a significant relationship between the attention allocation scores and self-reported motion sickness susceptibility. There was also no significant correlation between the measures derived from the SART task and experienced cybersickness.

Our inability to replicate the results by Wei et al. (2018) could be due to the differences in the measure of attentional allocation. In the original study, subjects performed the SART during coherent background motion that induces vection and also during incoherent motion. The incoherent motion condition was used as a baseline when computing the measure of attention allocation. In our Experiment 1, we only tested SART with the vection-inducing background motion, so our measure did not normalize for any differences in attention allocation during incoherent motion. If a tendency to attend to the periphery during vection is associated with decreased motion sickness susceptibility, either measure of attentional allocation would be expected to show a negative correlation. However, it is possible that our measure was less sensitive. To test whether the difference in measures could account for our failure to replicate Wei et al. (2018) in Experiment 1, we performed a closer replication in Experiment 2.

## Experiment 2

In this follow-up study, we modified the virtual reality exposure conditions to provide a stronger test of whether allocating attention to the central or peripheral visual field reduces cybersickness. For the virtual reality exposure, we implemented a stronger manipulation of visual attention allocation and also controlled the simulated player movement. A dot-probe task resembling the SART task was integrated into the virtual experience. Subjects “rode” a virtual rollercoaster ride that was expected to produce mild cybersickness. Throughout the exposure, dots were periodically presented in either the central or peripheral region of the display to encourage sustained attention to the CVF or PVF, depending on the experimental condition.

We also modified our method for measuring attention allocation during vection to more closely match the method used by Wei et al. (2018). Wei et al. (2018) tested the SART task with two types of background motion: coherently rotating dots or incoherently rotating dots. Our Experiment 1 only included the coherently rotating background. Experiment 2 tested both of the SART conditions, which allowed us to compute the same attention allocation measure used by Wei et al. (2018).

## Methods

### Preregistration

The methods and analysis plan were preregistered on the Open Science Framework (OSF) before data collection: https://osf.io/f7q8e. The only deviation was that we used log_10_(SSQ/10 + 1) to transform the SSQ scores rather than log_10_(SSQ + 10), which has no effect on the statistical test results. There were no other deviations from the preregistered plan for Experiment 2.

### Participants

Forty subjects participated in Experiment 2. Our sample consisted of 10 males and 30 females, with ages between 18 and 23 years old (*M* = 19.075, *SD* = 1.289). Subjects were recruited from the population of the University of Hong Kong and were paid either $100 HKD or course credits as compensation. We required all subjects to have normal or corrected-to-normal vision, with no history of vestibular or neurological functioning impairment.

The sample size was chosen to be able to reliably detect a mean difference in MISC score of 1 or more and also be able to replicate the main findings of Wei et al. (2018). The Misery Scale (MISC) is a 10-point self-administered scale. If our attention manipulation changes the mean MISC score by less than 1, it would have limited practical utility. Based on the variability of MISC scores observed in Experiment 1, a difference in MISC score of 1 would correspond to an effect size of *d*_*z*_ = 0.53. Our sample size provided 90% power to detect an effect size of *d*_*z*_ = 0.53 at an alpha level of .05. Our sample size also had at least 84% power to replicate the effect reported in Wei et al. (2018). Their study observed a correlation of *r* = − .479 between their measure of attention allocation based on the SART task (TRT) and the total motion sickness susceptibility measure (MSSQ-T).

### Apparatus and stimuli

Experiment 2 consisted of two tasks: the virtual reality exposure and the Sustained Attention to Response Task (SART) based on Wei et al. (2018).

#### Virtual reality exposure

For the VR exposure, subjects viewed a virtual rollercoaster ride for a maximum duration of 8 min. The virtual environment in Experiment 2 resembled a cartoon-like rural region, populated by various ruins, hills, campsites, trees, and bushes. The virtual scene was coded with Unity version 2019.4.38f1 and presented with an HTC Vive HMD. The environment was rendered at a resolution of 2160 × 1200 (1080 × 1200 per eye) at a 90 Hz refresh rate.

During the exposure, subjects were “seated” in a self-moving cart. They were given the ability to translate head movements to the virtual environment. The cart moved forward at a constant speed of 20 km/h, following an invisible rail track with multiple horizontal turns. Through the ride, subjects were presented with a white/black dot at a randomized 1500-2000 ms interval. Each target remained visible on the display for 500 ms. Subjects were instructed to press the “Shift” key when they detect a white dot, but do nothing for the black dots.

Two attention allocation conditions (Fig. 1—bottom row) were tested in a within-subjects design. For the central-cued condition, the white/black dots only appeared in the central region of the HMD display (FOV: 0°–5°). The experimenter verbally encouraged the subjects to pay more attention to the CVF in this condition, and a white transparent reference point was provided to indicate the center of the HMD display. For the peripheral-cued condition, the targets appeared in the peripheral region of the HMD display (FOV: 20°-30°) instead. No reference point was provided, and the subjects were told to attend more to their visual peripheral in this condition. The presentation order of the two attention allocation conditions was counterbalanced.

#### Sustained Attention to Response Task (SART)

The SART task in Experiment 2 was similar to the task used in Experiment 1, except that the 200 SART trials were divided into two blocks with either coherently rotating stimuli (CRS) or incoherently rotating stimuli (IRS). The CRS stimuli would be expected to induce vection, while the IRS stimuli would not.

Each block consisted of 100 trials (10 × Green-CVF, 10 × Green-PVF, 40 × Red-CVF, 40 × Red-PVF). Trials in the CRS block were identical to the ones in Experiment 1. For the IRS block, each gray dot had a different center of rotation, which were randomly distributed inside an invisible circular window (FOV: 0°–33.3°). The angular velocity in the IRS block was set to 24°/s to ensure that the mean rates of motion between the two blocks were similar.

### Measurements

The measures of motion sickness were the same as in Experiment 1. As in the previous experiment, the SSQ scores were log-transformed and normalized before statistical analyses, and the MISC scores used for analysis were taken from the exposure block that resulted in at least 75% completion.

In Experiment 2, we computed two measures of attention allocation tendency from the response times to CVF and PVF stimuli in the SART task:

$$ \Delta{{\text{RT}}}  =  {\text{RT}}_{\text{CVF,Coherent}} - {\text{RT}}_{\text{PVF,coherent}}$$

$$\begin{aligned} \Delta{{\text{RT}}}_{\text{norm}} & = ({\text{RT}}_{\text{CVF,Coherent}} - {\text{RT}}_{\text{CVF,Incoherent}}) \\& - ({\text{RT}}_{\text{PVF,Coherent}} - {\text{RT}}_{\text{PVF,Incoherent}}). \end{aligned}$$

The measure ΔRT is the difference between the median response time to CVF and PVF stimuli presented with a coherently moving background. This is the same measure used in Experiment 1. The measure ΔRT_norm_ is the difference in median response times to CVF and PVF stimuli after normalizing for the response times in the condition with incoherent background motion. This is equivalent to the attention allocation measure used by Wei et al. (2018). As in Experiment 1, we used the Hodges–Lehmann estimator to compute the median response times. For both measures, higher values indicate relatively more attention to the PVF during vection.

### Procedure

The overall procedure was the same as in the previous experiments. There were only two differences. In Experiment 2, subjects experienced passive simulated motion while performing a dot-probe task (see above), rather than performing a navigation task. In addition, Experiment 2 tested two types of SART trials, CRS and IRS, rather than just CRS. The two SART blocks were tested in separate sessions in counterbalanced order.

## Results

### Cybersickness during VR exposure

#### Virtual reality exposure completion rate

**Fig. 8:**
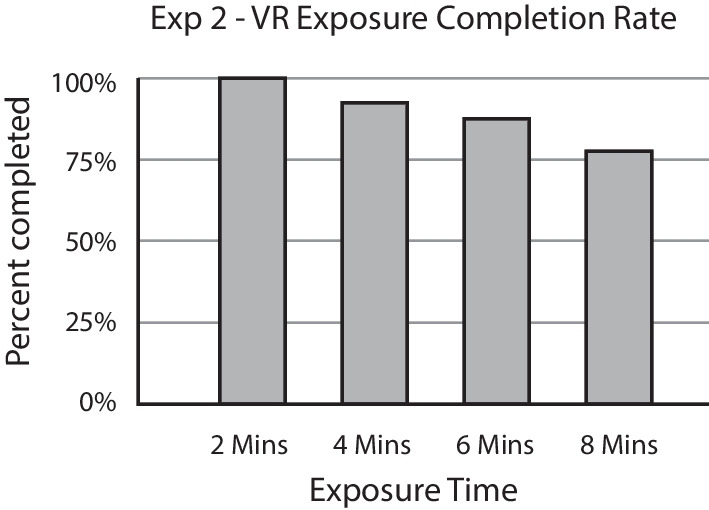
Percent of subjects who completed each of the VR exposure blocks in Experiment 2. The procedure was stopped if subjects reported more than mild motion sickness symptoms (>6 on MISC scale). Over 75% of the subjects were able to complete the full 8 min of exposure

The MISC scores used for analysis were chosen based on the completion rate, as in Experiment 1. Figure 8 plots the percentage of subjects who completed each exposure block. More than 75% of subjects (*n* = 31) were able to complete all four exposure blocks, so we used the MISC scores from each subject’s last completed block for analyses. For most subjects, this was the MISC score from the final exposure block.

#### Central-cued and peripheral-cued (H1 and H1a)

To test the main hypothesis of this study, we conducted paired-sample t tests to compare the mean cybersickness scores (MISC and SSQ) from the central-cued and peripheral-cued conditions. As in Experiment 1, statistical tests were performed on the raw MISC scores and the transformed and normalized SSQ scores.

**Fig. 9:**
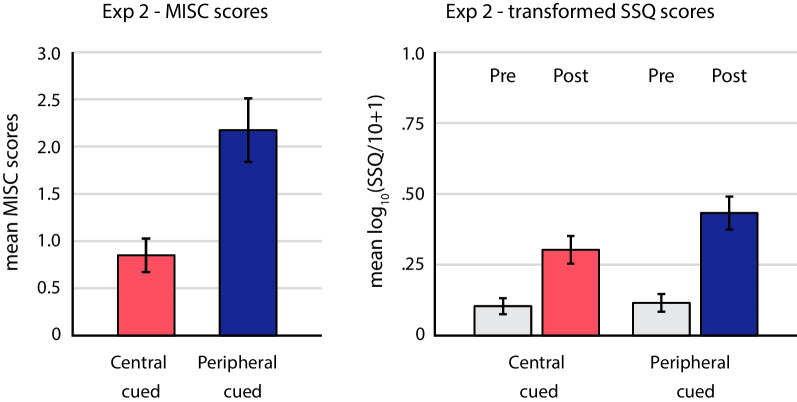
Motion sickness in the central-cued (red) vs peripheral-cued (blue) conditions of Experiment 2. The left graph plots mean MISC scores. The right graph plots mean SSQ scores after transforming by log_10_(SSQ/10 + 1). Pre-exposure SSQ scores are shown in gray. Error bars depict standard errors of the mean

The results revealed that cybersickness was lower in the central-cued condition than in the peripheral-cued condition. Figure 9 shows the mean MISC and transformed SSQ scores for the two attention allocation conditions. The mean MISC score in the peripheral-cued condition (*M* = 2.175, *SD* = 2.123) was significantly higher than in the central-cued condition (*M* = 0.85, *SD* = 1.122), *t*(39) = 4.38, *p* < .001, *d*_*z*_ = 0.693. For the SSQ scores, the same pattern was observed: The mean change in log_10_(SSQ-T/10 + 1) was significantly larger in the peripheral-cued condition (*M* = 0.318, *SD* = 0.280) than in the central-cued condition (*M* = 0.199, *SD* = 0.268), *t*(39) = 3.302, *p* = .002, *d*_*z*_ = 0.522. Further analyses revealed a consistent pattern across all SSQ subscales, with all three subscales showing significantly higher cybersickness in the peripheral-cued condition (nausea: *t*(39) = 3.133, *p* = .003, *d*_*z*_ = 0.495; oculomotor disturbances: *t*(39) = 2.874, *p* = .007, *d*_*z*_ = 0.454; disorientation: *t*(39) = 2.289, *p* = .028, *d*_*z*_ = 0.362). Contrary to Wei et al. (2018), our findings demonstrate that encouraging attention to the PVF during VR exposure leads to more cybersickness than focused attention to the CVF (supporting H1a).

#### Bayesian analysis for H1 and H1a

We performed additional Bayesian analyses to estimate credible intervals for the differences between cybersickness in the attentional cueing conditions. For each measure, we estimated the posterior distribution for differences using Bayes theorem, and then computed the 95% highest density intervals (HDIs) of credible values. Noise was assumed to be normally distributed. We used non-informative priors for the unknown parameters: a uniform distribution for mean difference and a Jeffreys prior for the variance. The posterior distribution for mean differences was computed by numerically integrating over the variance parameter: P(m|{xi}) ~  ∫P({xi}|m,v) (1/v) dv. Using the estimated posterior, we determined the 95% highest density interval (HDI), which is the smallest interval containing 95% of the posterior probability. For the SSQ, the HDIs were computed from the normalized and log-transformed data and then converted back to the original scale.

The results indicate that attentional cueing produced relatively large changes in experienced cybersickness. The estimated 95% HDI for the difference between the MISC score in the peripheral-cued and central-cued attention conditions was [0.718, 1.941]. Relative to the mean MISC in the peripheral-cued condition (2.18), the lower bound for the size of the difference would be a 33% reduction in cybersickness in the central-cued condition, and the upper bound would be a 90% reduction. For the SSQ data, the 95% HDI for the differences between log_10_(SSQ-T/10 + 1) in the peripheral-cued and central-cued conditions was [0.0453, 0.1901]. We converted these differences to differences in raw SSQ total scores using the mean post-test SSQ in the peripheral-cued condition (16.7) as a baseline, and the resulting 95% HDI for the difference in SSQ scores was [− 2.31, − 8.36]. Based on these results, the central-cued condition produced a 16–57% reduction in cybersickness as measured by the SSQ total. For both measures, the lower bounds for the size of the effects would correspond to a meaningful change in cybersickness.

### Attention allocation tendency during vection

#### Attention allocation tendency and MSSQ-short (H2 and H2a)

We performed correlation analyses to examine the relationship between attention allocation during vection and self-reported motion susceptibility (H2 and H2a). We tested two measures derived from SART performance: ΔRT, which was the measure used in Experiment 1, and ΔRT_norm_, which was used by Wei et al. (2018). Based on the results of Wei et al. (2018), both attention allocation measures would be expected to be negatively correlated with the MSSQ-Short scores.

**Fig. 10:**
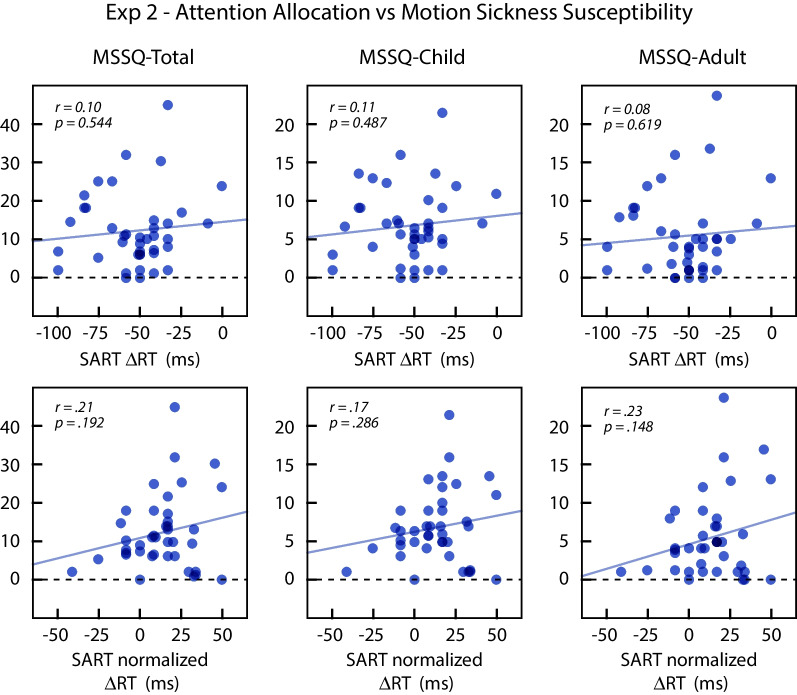
Self-reported motion sickness susceptibility (MSSQ) of individual subjects in Experiment 2 plotted as a function of their attentional allocation scores derived from SART. The two rows use different measures of attentional allocation: the mean difference in reaction times for central and peripheral targets with coherent background motion (top row), or the mean difference after normalizing for reaction times in the incoherent motion conditions (bottom row). Positive values of ΔRT correspond to more attention to the periphery. Lines show the regression fits. The left graph plots the total MSSQ scores, and the middle and right graphs plot the MSSQ child and MSSQ adult subscores

The results did not reveal any significant relationship between attention allocation and MSSQ-Short scores, regardless of the attentional allocation measure. Figure 10 plots the three MSSQ-Short measures as a function of ΔRT (top row) and ΔRT_norm_ (bottom row). There was no significant correlation between ΔRT_norm_ and the MSSQ-Short total scores (*r*(38) = .211, *p* = .192), child subscores (*r*(38) = .173, *p* = .286), or adult subscores (*r*(38) = .233, *p* = .148). Similarly, there was no significant correlation between ΔRT and MSSQ-Short total scores (*r*(38) = .099, *p* = .544), child subscores (*r*(38) = .113, *p* = .487), or adult subscores (*r*(38) = .081, *p* = .619). We were unable to replicate the negative relationship between attention allocation tendency during vection and self-reported motion sickness susceptibility scores as reported by Wei et al. (2018), and the small trends were in the opposite direction.

#### Bayesian analysis for H2 and H2a

We performed an additional Bayesian analysis to estimate 95% confidence intervals of credible values for the correlation between SART score and motion sickness susceptibility. The posterior distributions were estimated using JASP, with the default assumption of a uniform prior for the correlation coefficient.

When using the measure of Wei et al. (2018) as an indicator of attention allocation tendency (∆RT_norm_), the 95% HDIs for correlations between SART and MSSQ-Short scores were [− .105, .475] for MSSQ total, [− .142, .445] for MSSQ child, and [− .083, .493] for MSSQ adult. Using the non-normalized measure of attention allocation (∆RT), the 95% HDI for the correlation with MSSQ total score was [− .212, .385]. For the subscales, the 95% credible intervals were [− .199, .397] for the MSSQ child subscore, and [− .228, .370] for the MSSQ adult subscore.

Based on these results, any correlation between attention allocation during vection and motion sickness susceptibility in the direction predicted by Wei et al. (2018) was small, |*r*|< 0.23. The correlations reported by Wei et al. (2018) were *r* = − .479 for the SART and MSSQ total scores and *r* = − .595 for the SART score and MSSQ adult subscores. Both of these are outside the corresponding 95% HDIs from our data. The correlation between SART and MSSQ child subscore was not reported by Wei et al. (2018), as it was not significant.

#### Attention allocation tendency and cybersickness (H3 and H3a)

We performed correlation analyses to examine the relationship between attention allocation tendency during the SART task and cybersickness experienced during the VR exposure (H3 and H3a). As in Experiment 1, we computed overall measures of cybersickness by averaging the post-exposure MISC or SSQ scores from the central-cued and peripheral-cued conditions.

**Fig. 11:**
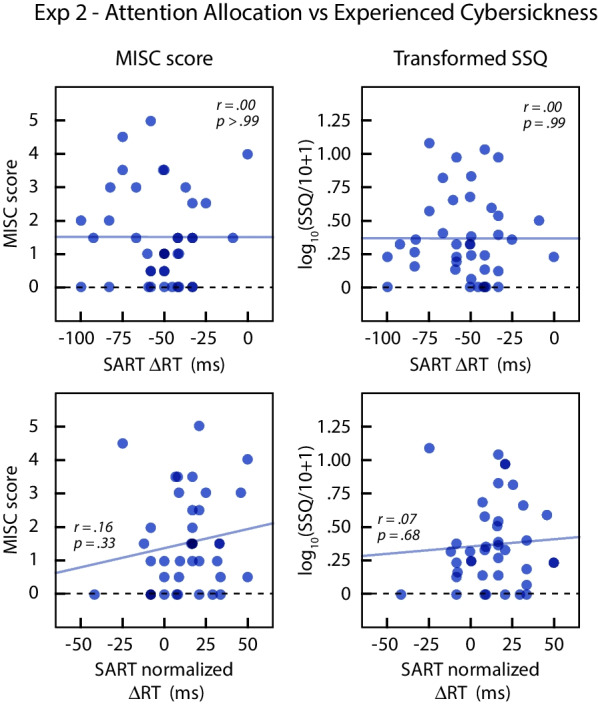
Cybersickness experienced by individual subjects during virtual navigation in Experiment 2 plotted as a function of their attentional allocation scores. The two rows use different measures of attentional allocation: the mean difference in reaction times for central and peripheral targets with coherent background motion (top row), or the mean difference after normalizing for reaction times in the incoherent motion conditions (bottom row). Positive values of ΔRT correspond to more attention to the periphery. Lines show the regression fits. The left graph plots the MISC scores and the right graph plots the SSQ scores after transforming by log_10_(SSQ/10 + 1)

Figure 11 shows overall MISC and SSQ scores as a function of ∆RT (top row) and ∆RT_norm_ (bottom row). The results were almost the same for the two attention allocation measures. There was no significant correlation between ∆RT and overall MISC (*r*(38) = − .001, *p* = .996) or overall SSQ (*r*(38) = − .001, *p* = .993). There was also no correlation between ∆RT_norm_ and overall MISC (*r*(38) = .158, *p* = .332) or overall SSQ (*r*(38) = .067, *p* = .680). Overall, there did not appear to be any relationship between the attention allocation tendency during SART and the cybersickness experienced during the virtual reality exposure.

#### Bayesian analysis for H3 and H3a

We also performed a Bayesian analysis to estimate 95% credible intervals for the correlation between SART scores and motion sickness. The posteriors and HDIs were computed using JASP and assuming a uniform prior.

For the MISC scores, the 95% HDI for the correlation with ∆RT was [− .302, .300], and with ∆RT_norm_ it was [− .157, .433]. For SSQ, the 95% credible interval for the correlation with ∆RT was [− .302, .300], and with ∆RT_norm_ it was [− .241, .359]. If there was correlation between SART performance and experienced cybersickness, our results indicate that the correlation was likely to be within [− .302, .433].

### MSSQ-short and cybersickness

**Fig. 12:**
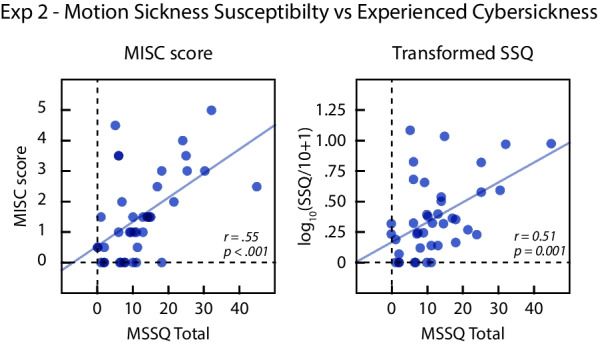
Cybersickness experienced by individual subjects in Experiment 2 plotted as a function of self-reported motion sickness susceptibility (MSSQ total). Lines show the regression fits. The left graph plots the MISC scores and right graph plots the SSQ after transforming by log_10_(SSQ/10 + 1)

We performed additional exploratory analyses to assess whether MSSQ-Short is an effective predictor of cybersickness experienced in our VR exposure conditions. Figure 12 plots the relationship between MSSQ-Short total scores and the overall cybersickness experienced during the exposure. Consistent with the results from Experiment 1, we observed a significant positive correlation between MSSQ-Short total scores and overall MISC scores (*r*(38) = .536, *p* < .001), as well as MSSQ-Short total scores and overall SSQ scores (*r*(38) = .537, *p* < .001). Taken together, our results suggest that the MSSQ-Short does have utility for predicting individual differences in susceptibility to cybersickness.

## Discussion

### Manipulation of visual attention and cybersickness

Experiment 2 implemented a dot-probe task to encourage sustained attention to either the CVF or PVF during VR exposure. With this manipulation, we detected a significant difference in cybersickness between the attention-cueing conditions. The observed difference provides evidence that the dot-probe task did affect attention allocation.

Encouraging subjects to focus more on their central vision resulted in comparatively less cybersickness than when attention to the periphery was encouraged. This difference was observed in all of the cybersickness measures—MISC, SSQ, and SSQ subscales. This finding contradicts the prediction made by Wei et al. (2018) that attention to the periphery would reduce cybersickness. On the other hand, it is consistent with previous studies that have found that restricting the FOV reduces cybersickness.

The dot-probe task in the central-cued and peripheral-cued conditions might have differed in difficulty, but it is unlikely that this explains the observed difference. Previous studies suggest that an increase in cognitive load can lead to a decrease in motion sickness (Bos, 2015; Seno et al., 2011). As individuals become more mentally occupied in sickness-inducing situations, such as attempting to solve a challenging math question during virtual navigation, this may distract them away from the experienced symptoms. If there was a difference in task difficulty in our conditions, the peripheral-cued condition would be the more challenging task because there were more variations in the target locations. The peripheral-cued conditions would therefore be expected to produce less cybersickness, which is in the opposite direction as the difference observed in Experiment 2. Sepich et al. (2022) reported that excessive cognitive load might instead elevate cybersickness. However, we believe that neither the central-cued nor peripheral-cued version of the dot-probe task was overly demanding to our subjects, so a benefit of increased cognitive load on cybersickness would still be expected. Although cognitive load might have had some effect on cybersickness, it is unlikely that this was the cause of the reduced cybersickness with central attention in our experiment.

It should be noted that our current findings may have underestimated the actual effectiveness of our manipulation in mitigating cybersickness due to a potential floor effect. In Experiment 2, there were a sizeable number of subjects who appeared to be highly resilient to cybersickness. At least a quarter of our sample (*n* = 10) did not experience any discomfort in either of the attention-cueing conditions as measured by MISC ratings. For users who are naturally not susceptible to cybersickness, any sickness-reducing intervention would have little impact. The estimated effect size observed in Experiment 2 was *d*_*z*_ = 0.362 − 0.693, depending on the measure. If our sample had been composed entirely of cybersickness-susceptible individuals, the effect size would likely have been larger.

### Visual attention allocation and motion sickness susceptibility

In Experiment 2, we were still unable to replicate the findings of Wei et al. (2018) despite using the same measures and a larger sample size that had sufficient power. For both ∆RT (the attention allocation measure used in Experiment 1) and ∆RT_norm_ (the attention allocation measure used by Wei et al. (2018)), attention allocation as measured by the SART was not predictive of self-reported motion sickness susceptibility. Even using the same measures as Wei et al. (2018) and a more closely matched design, we did not replicate their main finding.

The results from our Bayesian analysis indicate that any correlation between attention allocation and motion sickness susceptibility in the expected direction was small. The lower bound of the 95% interval of credible values was *r* = − .228, indicating that any negative relationship that was present explained at most 5.2% of the variance. If we missed a negative correlation that was present, the size of the effect is likely to be minimal.

We also found no relationship between baseline attention allocation and experienced cybersickness. Null results were observed for both of the attention allocation measures (∆RT and ∆RT_norm_). The results from the Bayesian analyses indicate that any correlation between attentional allocation and cybersickness was unlikely to have a correlation of *r* > .433 or *r* < − .302. We cannot rule out the possibility that a medium-sized correlation (|*r*|= 0.3–0.4) was present but missed.

## General discussion

Across experiments, we found no evidence that allocating attention to the periphery reduces cybersickness (H1). Based on the findings of Wei et al. (2018), we hypothesized that reallocating visual attention to the PVF during VR exposure would reduce experienced cybersickness. A negative correlation between attention allocation tendency and overall experienced cybersickness would also be expected (H3). Neither of these effects was observed. We also failed to replicate the negative correlation between attention allocation during vection and self-reported motion sickness susceptibility reported by Wei et al. (2018) (H2).

Instead, we found evidence to support our main alternative hypothesis (H1a). Given that previous studies found that restricted FOV can reduce cybersickness, we hypothesized that encouraging attention to the CVF, rather than the PVF, would reduce cybersickness experienced in VR. Consistent with this hypothesis, Experiment 2 found that cybersickness was lower in the central-cued condition compared to the peripheral-cued condition. A further prediction would be that attentional allocation tendency would be positively correlated with motion sickness susceptibility (H2a) and overall cybersickness (H3a). These hypothesized correlations were not observed in either experiment.

### Manipulating visual attention to reduce cybersickness

We manipulated visual attention allocation to CVF/PVF using task-relevant visual cues (Experiment 1) or a dot-probe task (Experiment 2). Experiment 1 did not find any significant difference between the two attention-cueing conditions, but Experiment 2 found that cybersickness was reduced in the central-cued condition.

One possible reason that Experiment 1 did not find a detectable difference between the two attention-cueing conditions is that the peripheral-cued condition did not produce sustained visual attention to the periphery. In Experiment 1, we used exogenous visual cues (i.e., highlighting the chests in red) to manipulate attention allocation in VR, which served as hints about the location of the chests. Because the chests were distributed across the environment, the cues would generally encourage a wide allocation of attention. However, after being cued to the location of a chest, subjects might quickly shift their attention to focus on a single target chest, rather than maintaining a wide allocation of attention. If the exogenous cues in the peripheral-cued conditions had only transient effects on attention, then this could have prevented any effects on cybersickness.

Another factor that could have contributed to the null finding in Experiment 1 is differences in experienced motion. We allowed subjects to freely control their virtual movement. Although we did not record the virtual movement in Experiment 1, we noticed that some subjects performed less active navigation as they experienced discomfort, which may have been a strategy to reduce cybersickness. If subjects altered their movement in response to discomfort, this would tend to reduce any differences between conditions.

Experiment 2 was designed to avoid the limitations of Experiment 1. The dot-probe task provided a more reliable manipulation of attentional allocation. To perform well, subjects would have to continuously attend to either the CVF or PVF during the VR exposure. The observed difference in cybersickness confirms that the manipulation did have some effect. Experiment 2 also controlled the simulated motion across conditions. Subjects were “seated” in a self-driving cart during VR exposure, and thus unable to slow down or pause in-game movements when they begin to experience symptoms. These methodological differences could explain why an effect of attentional cueing on cybersickness was observed in Experiment 2 but not in Experiment 1.

A connection can be drawn between the effect of attention reallocation and FOV on cybersickness. Previous studies have attempted to control cybersickness by reducing the in-game FOV (Lin et al., 2002; Rebenitsch & Owen, 2016; Saredakis et al., 2020; Weech et al., 2019). The sensory conflict is minimized as restrictive FOV removed the motion cues available in the visual periphery. In Experiment 2, although periphery motion cues remain available across the two conditions, the central-cued condition encouraged the subjects to constantly attend to their CVF, which would limit the attentional resources that are available for peripheral motion cues. For both types of interventions, CVF attention cueing and restrictive FOV, lowered cybersickness could be explained by reduced processing of peripheral motion cues.

### Baseline attention allocation tendency and motion sickness

In both experiments, we failed to replicate the findings by Wei et al. (2018). No significant correlation was observed between motion sickness susceptibility scores and the two attention allocation measures (∆RT or ∆RT_norm_).

The negative relationship between attention allocation tendency during vection and motion sickness susceptibility reported by Wei et al. (2018) may not reliable, or might have been a false positive. In Experiment 1, the failure to replicate might have been due to insufficient power and some methodological differences. However, Experiment 2 was a closer replication and well-powered but still did not replicate the previous findings. We were unable to detect any significant relationship between MSSQ-Short and attention allocation tendency as measured by either ∆RT (the measure used in Experiment 1) or ∆RT_norm_ (the measure used in Wei et al. (2018)). Furthermore, the results from Experiment 2 indicate that any negative relationship between attentional allocation tendency and motion sickness susceptibility was very small: *r* > − .228 for ∆RT and *r* > − .142 for ∆RT_norm_.

We also did not observe any correlation between attention allocation during vection and overall experienced cybersickness, which appears to conflict with the experimental results from Experiment 2. In Experiment 2, we found that cybersickness was reduced in the central-cued condition. Given this finding, one might expect that people with a natural allocation tendency toward the CVF would be less likely to experience motion sickness during VR exposure, which was not observed.

It is possible that the baseline level of attention allocation is not representative of the attentional distribution when performing tasks in virtual reality. Allocation of attention is known to be flexible and can be shifted with various visual cues (Kean & Lambert, 2003). Therefore, any context-relevant visual cues that manipulate visual attention allocation, such as performing a dot-probe task in VR or receiving an in-game notification on a newly received side quest, could disrupt the effect of the default attention allocation tendency. This could explain why the default attention allocation tendency was not predictive of experienced cybersickness in our studies, despite the fact that our manipulation of attention affected cybersickness in Experiment 2.

### Effectiveness of MSSQ-short in predicting cybersickness

We performed exploratory analyses to assess the effectiveness of MSSQ-Short in predicting cybersickness. Although both experiments found positive correlations between MSSQ-Short and overall MISC/SSQ scores, the amount of correlation was modest: *r* = .448 − .558. Anecdotally, we observed that multiple subjects rated themselves as highly susceptible to motion sickness with the MSSQ-Short but experienced minimal cybersickness during the actual exposure. This raises the question of whether the MSSQ-Short is a valid predictor of cybersickness susceptibility.

It has been suggested that the MSSQ may not be the optimal tool for predicting visually induced motion sickness, such as motion sickness induced by VR exposure. Keshavarz et al. (2019) pointed out that the MSSQ was not specifically constructed to gauge one’s likelihood of experiencing visually induced motion sickness, but rather symptoms that are induced physically. The MSSQ derives general motion sickness susceptibility from one’s history of discomfort in situations where physical motion is expected, such as riding a boat. None of the MSSQ items capture the incidence of motion sickness symptoms in situations where strong physical motion is unlikely to occur, such as watching a 3D movie. To accurately predict cybersickness, alternative instruments that are specifically designed to measure visually induced motion sickness susceptibility specifically might be preferred.

## Conclusion

We discovered that reallocating attention to the central visual field can reduce cybersickness symptoms. This finding was observed in Experiment 2, which was a well-powered experiment that avoided some methodological limitations of Experiment 1.

The effect of allocating attention to the CVF on cybersickness may be comparable to the effect of restricting FOV. Previous studies have found that restricting FOV can reduce cybersickness (Lin et al., 2002; Rebenitsch & Owen, 2016; Saredakis et al., 2020; Weech et al., 2019). Our results demonstrate that restricting attention to the CVF can also reduce cybersickness. Both interventions might reduce the severity of cybersickness by minimizing the processing of peripheral motion cues.

We found no evidence that attention allocation tendency predicts self-reported susceptibility to motion sickness or experienced cybersickness during VR exposure. Experiment 2 failed to replicate the findings of Wei et al. (2018) despite using a closely matched method and having good statistical power. Because visual attention can be easily shifted with context-relevant visual cues, the default attention allocation tendency may not be useful for predicting cybersickness.

## Data Availability

The data and materials are available at the public repository: https://osf.io/8cbw9/

## Declarations

### Preregistration

The experiments were both preregistered on the Open Sciences Framework, Experiment 1: https://osf.io/rfzwt, Experiment 2: https://osf.io/f7q8e.
