# Correction: Restricting the distribution of visual attention reduces cybersickness

**DOI:** 10.1186/s41235-023-00491-0

**Published:** 2023-05-29

**Authors:** Sai Ho Yip, Jeffrey Allen Saunders

**Affiliations:** Department of Psychology, University of Hong Kong, Hong Kong, Hong Kong

**Correction: Cognitive Research: Principles and Implications (2023) 8:18** 10.1186/s41235-023-00466-1

The original article [1] contained an error whereby the production team handling the article erroneously removed an equation on Page 11 after proofing. The equation has since been re-instated.
